# Short-term MRI Follow-up and Thin-layer PDWI Sequence without Fat Suppression for Detecting Cartilage Loose Bodies: A Case Report

**DOI:** 10.2174/0115734056354843250321170055

**Published:** 2025-04-09

**Authors:** Ying Liu, Lei Gao, Junfei Li, Jujia Li, Jian Zhao

**Affiliations:** 1 Department of Medical Imaging, Hebei Medical University Third Hospital, Qiaoxi District, Shijiazhuang 050051, Hebei, China

**Keywords:** Loose body, Osteochondritis dissecans, Cartilage fracture, Knee, Microfracture surgery, Magnetic Resonance Imaging

## Abstract

**Background:**

Osteochondritis Dissecans (OCD) is an idiopathic process and can progress from stable to cartilage fragmentation with the formation of loose bodies in the affected joint capsule. Loose bodies in the knee may wear out the articular cartilage, tendons, and ligaments, leading to a series of problems, such as joint locking, bouncing, joint effusion, and meniscus tear; therefore, early recognition and treatment of intraarticular loose bodies are important to achieve favorable long-term outcomes.

**Case Report:**

A 49-year-old male presented with a 1-month history of right knee discomfort. The patient underwent a knee MRI scan and was diagnosed with OCD. A short-term MRI follow-up with a thin-layer PDWI sequence without fat suppression detected the cartilage fragments in the knee capsule. Loose body removal, cartilage repair, and microfracture surgery were performed under arthroscopic surgery, and loose bodies of cartilage fragments were removed.

**Conclusion:**

Short-term MRI follow-up and thin-layer PDWI sequence without fat suppression are necessary for detecting cartilage loose bodies.

## 1 INTRODUCTION

Osteochondritis Dissecans (OCD), first described in 1888 by the German surgeon Franz König, also known as an osteochondral lesion, is not fully understood. However, it is believed to be multi-factorial in etiology [1]. OCD is an idiopathic process and can occur from childhood through adult life, with the majority of patients presenting in their teenage years. The lesions can progress from a stable state to fragmentation of the overlying cartilage with the formation of a loose body in the affected joint capsule [2].

Intraarticular loose bodies refer to objects that appear in the joint capsule for various reasons [3]. According to their texture, they can be divided into bony, cartilaginous, fibrous, *etc*. Magnetic Resonance Imaging (MRI) has the advantage of high contrast of soft tissue, multi-parameters, and multi-dimensional imaging, which can detect the composition of loose bodies [4]. MRI also provides a clear and stereoscopic display of the location and number of intraarticular loose bodies, subchondral bone and ligament, and meniscus [5].

Loose bodies in the knee may wear out the articular cartilage, tendons, and ligaments, leading to a series of problems, such as joint locking, bouncing, joint effusion, and meniscus tear; therefore, early recognition and treatment of intraarticular loose bodies are important to achieve favorable long-term outcomes. This case report introduces the short-term clinical and radiographic follow-up of a patient with knee joint OCD and explores the value of short-term MRI follow-up and thin-layer proton density-weighted image (PDWI) in detecting the loose bodies of knee joints.

## 2 CASE REPORT

**Fig. (1):**
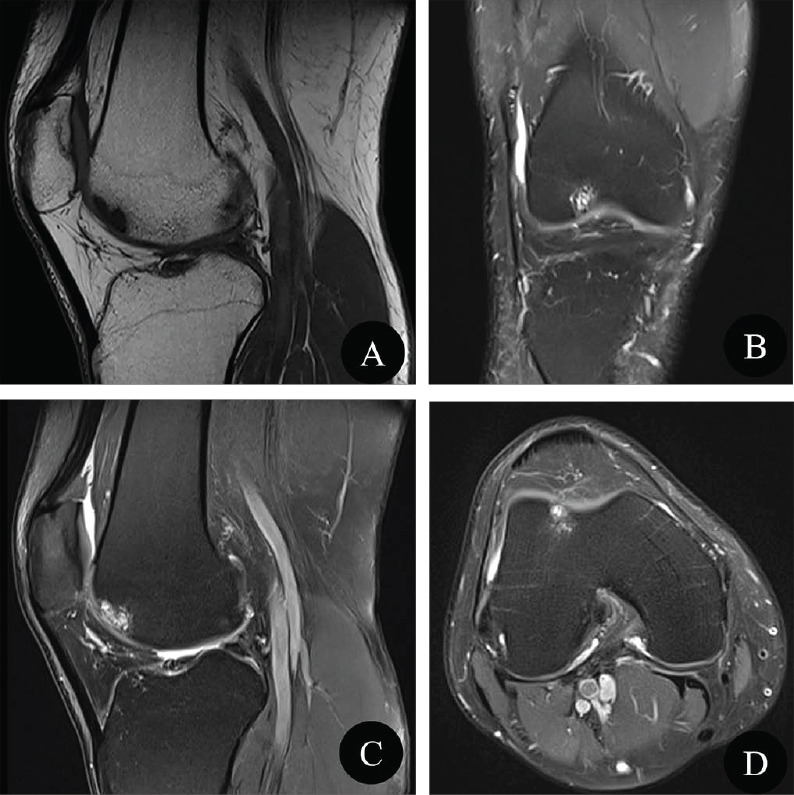
Initial presentation of the right knee MRI examination. Sagittal T1-weighted image (**A**, T1WI), coronal fat-suppressed proton density-weighted image (**B**, PDWI-fs), sagittal PDWI-fs (**C**), and axial PDWI-fs (**D**) showed the unstable OCD lesion of the lateral femoral condyle. MRI images showed that the local articular cartilage of the lateral femoral condyle was thinner and heterogeneous, had slightly higher signal intensity on the PDWI-fs image, and was surrounded by multiple small cysts in the subchondral bone.

A 49-year-old, right-hand-dominant male presented to the Orthopedic Department with a 1-month history of right knee discomfort. He had a history of right knee joint trauma for 4 years and did not have hypertension, diabetes, or coronary heart disease. MRI included sagittal T1-weighted Image (T1WI), sagittal fat-suppressed PDWI Image (PDWI-fs), axial PDWI-fs, and coronal PDWI-fs, and the parameters of each sequence were as follows (Table **1**). MRI images showed that the local articular cartilage of the lateral femoral condyle was thinner and heterogeneous. It had slightly higher signal intensity on the PDWI-fs image and was surrounded by multiple small cysts in the subchondral bone (Fig. **1**).

**Fig. (2):**
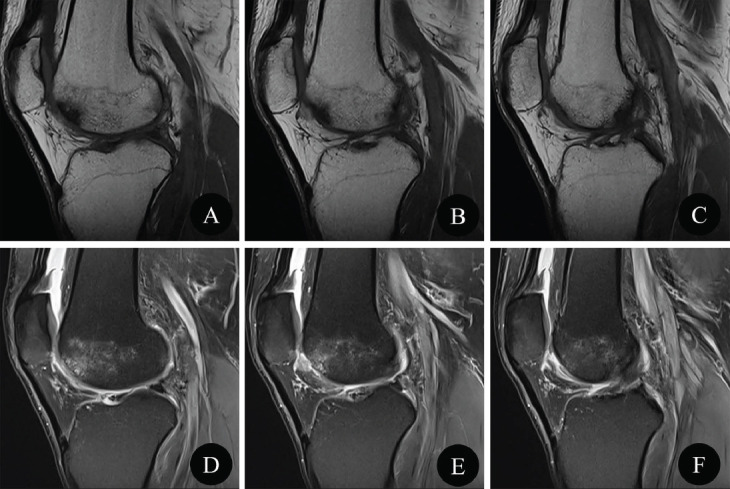
Second presentation of the right knee MRI examination. Sagittal T1WI (**A**-**C**) and PDWI-fs (**D**-**F**) showed local cartilage fracture and defect of the lateral femoral condyle, subchondral bone marrow edema, and joint effusion, while no cartilage loose body was clearly detected.

One month later, due to the aggravation of pain and discomfort in the right knee joint, occasionally joint locking, he was referred to the orthopedics department again, during which he played football intermittently. The patient underwent an MRI examination once more, and the MRI scan sequence and parameters were the same as 1^st^ MRI examination (Table **1**). MRI showed local cartilage fracture and defect of the lateral femoral condyle, subchondral bone marrow edema, and joint effusion, while no cartilage loose body was clearly detected (Fig. **2**). The diagnosis was Osteochondritis Dissecans (OCD, grade IV), and the treatment of arthroscopic surgery was suggested; however, the patient refused.

**Fig. (3):**
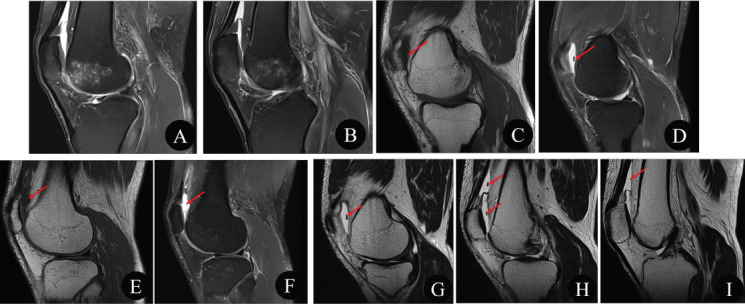
Three days after the second presentation, the third presentation of the right knee MRI examination. Sagittal PDWI-fs (**A**-**B**) showed that the area of subchondral bone marrow edema increased compared to three days ago, and the cartilage fracture and joint capsule effusion were basically the same. Sagittal T1WI (**C** and **E**) and PDWI-fs (**D** and **F**) showed two loose bodies (red arrows). The sagittal PDWI sequence of 2mm slice thickness without fat suppression (**G**-**I**) showed four loose bodies in the joint capsule (red arrows).

**Fig. (4):**
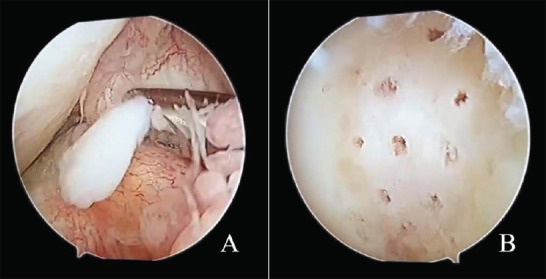
The patient underwent arthroscopic surgery for treatment. Loose body (**A**) removal, cartilage repair, and microfracture surgery (**B**) were performed under the arthroscope, and three loose bodies of cartilage fragments were removed.

Three days later, an MRI examination was performed again. The area of subchondral bone marrow edema increased compared to three days ago, and the cartilage fracture and joint capsule effusion were basically the same (Fig. **3A, B**). MRI showed two loose bodies, which were difficult to distinguish from the infra-patellar fat pad and synovial wrinkle (Fig. **3C-F**, red arrows). Additional sagittal PDWI sequence of 2mm slice thickness without fat suppression was performed, and other parameters were the same as sagittal PDWI-fs, as shown in Table **1**. Four loose bodies (Fig. **3G-I**, red arrows) were visible in the joint capsule on sagittal PDWI sequence of 2mm slice thickness without fat suppression, which were easy to distinguish from the infra-patellar fat pad and synovial wrinkle. The patient underwent arthroscopic surgery for treatment. Loose body removal, cartilage repair, and Microfracture Surgery (MFS) were performed under the arthroscope, and three loose bodies of cartilage fragments were removed (Fig. **4**).

The patient stated that a history of knee trauma was an important cause of OCD, and vigorous exercise was the main cause of unstable OCD, progressing to loss of bodies of articular cartilage. Short-term MRI follow-up and thin-layer PDWI sequence without fat suppression are necessary for detecting cartilage loose bodies. The first MRI examination found that the articular cartilage of the lateral femoral condyle was thinner, and there were multiple small cysts in the subchondral bone, which were not taken seriously. However, after playing football, the pain and discomfort in the right knee joint intensified. The second MRI examination diagnosed with OCD without loose bodies, while he refused the arthroscopic treatment recommendation due to personal reasons and fear of surgery. The third MRI examination clearly found loose bodies, and the doctor analyzed the risk of damage to the meniscus and ligaments, so he agreed to get the surgical treatment done. After the arthroscopic treatment, the joint was painful, swollen, and difficult to move in the first month, and 6 months later, the joint occasionally had a slight discomfort, but the symptoms of joint locking disappeared, and the pain of the knee joint was relieved.

## 3 DISCUSSION

OCD is categorized as a form of osteonecrosis affecting the subchondral bone, causing a piece of the subchondral bone and articular cartilage to detach from the underlying bone [6]. The ensuing fragment may be either stable, indicating intact overlying articular cartilage, or it may be unstable, which has significant prognostic implications [6]. Although OCD has been recognized for more than a century, its exact cause remains unclear [7]. Despite the lack of evidence of an inflammatory component in histopathology, the term “osteochondritis” has persisted over the years [8].

In order to meet the needs of clinical treatment and better observe the edema caused by the injury of the knee ligament, meniscus, and tendon, the conventional knee MRI scanning protocol includes four sequences: sagittal T1WI, sagittal PDWI-fs, axial PDWI-fs, and coronal PDWI-fs, and the slice thickness of these sequences ranging from 3 to 4mm as shown in Table **1**. There are four MR imaging criteria for OCD instability: (a) a high signal intensity rim surrounding an OCD lesion on T2-weighted images (hereafter, “high T2 signal intensity”), (b) cysts surrounding an OCD lesion, (c) a high T2 signal intensity fracture line extending through the articular cartilage overlying an OCD lesion, and (d) a fluid-filled osteochondral defect [9]. At the first presentation, an MRI showed that there were multiple small cysts in the subchondral bone of the lateral femoral condyle, indicating an unstable OCD lesion.

At the second presentation, an MRI examination of the right knee showed local cartilage fracture and defect of the lateral femoral condyle, while no clear cartilage loose bodies were observed. Although MRI has the advantage of observing soft tissues from multiple parameters and angles, there was a degree of difficulty in detecting cartilage fragments and loose bodies in the present case based on the conventional knee MRI scanning protocol. During this period, the patient played football, and football is a high-intensity exercise. A previous study indicated that mechanical overload played an important role in the pathogenesis of OCD [10]. We speculated that the loose cartilage bodies of the patellar may be wrapped by the infra-patellar fat pad or located in the suprapatellar capsule outside the scan field.

A thinner PDWI sequence without fat suppression is particularly effective in detecting loose bodies. At the third presentation, an MRI examination of the right knee showed only two loose bodies in the conventional knee MRI scanning protocol, with slightly higher signal on T1WI and low signal on PDWI-fs, which were difficult to distinguish from the infra-patellar fat pad and synovial wrinkle. After observing a supplementary sagittal PDWI sequence of 2mm slice thickness without fat suppression, four intraarticular loose bodies were clearly visible in the joint capsule. These bodies appeared with a low signal on PDWI without fat suppression image, making them easy to distinguish from the infra-patellar fat pad and synovial wrinkle. Infra-patellar fat pad showed a high signal on the PDWI image without fat suppression, and due to the thinness of the layers, cartilage loose bodies could be separated from the synovial wrinkle, and the discontinuity between the loose bodies and the synovial plicae could be found.

Moreover, a thinner PDWI sequence without fat suppression can be applied to other joints for carefully observing loose bodies in other joints and conditions of different patient populations.

In this case, the patient underwent arthroscopic surgery, cartilage repair, and MFS surgery, which were performed under the arthroscope, and three loose bodies of cartilage fragments were removed. In the T2 study of knee cartilage, a lower T2 value was found in MFS-induced fibrocartilage compared to hyaline cartilage [11]. Since one of the loose bodies in the suprapatellar capsule was about 1mm in size, it was too small to be clearly found under arthroscopy. Gold-standard for detecting loose cartilaginous bodies in the knee joint should be arthroscopy following high quality MRI. Although the patient refused to undergo arthroscopy, using MRI to follow up on joint loose bodies is very expensive and inefficient. Arthroscopic MFS is a treatment for cartilage defect repair [12], which can stimulate bone marrow and induce clot formation that typically evolves into fibrocartilage [13]. MFS is simple and minimally invasive, suitable for patients with unstable OCD, but the quality of regenerated cartilage may be slightly inferior, and there is a risk of osteoarthritis complications. Other treatment options for OCD, like refixation of cartilage fragments, autologous or homologous transplantation of chondral and osseous parts, and tissue engineering of cartilage (see also “nose to knee” transfer of tissue engineering cartilage), have their own advantages and disadvantages. Refixation of cartilage fragments can directly refix the fragments and promote cartilage healing; however, it may have limited effect on severely displaced fragments and is operationally complex [14]. Autologous or homologous transplantation of chondral and osseous parts has good tissue compatibility, good repair effect, and reduced pain, but it can cause donor site damage [15, 16]. Tissue engineering of cartilage (see also “nose to knee” transfer of tissue engineering cartilage) can customize the repair of cartilage defects, with good biocompatibility and mechanical properties; however, it is still in the research and experimental stage, and its long-term effects and safety need further observation [17]. The treatment of OCD requires a comprehensive consideration of the specific conditions of the patient, including age, severity of the condition, activity level, and other factors, to select the most appropriate treatment method.

## 3.1 Strengths and Limitations

This case report had some strengths: (1) the patient was followed up shortly with an MRI examination when the MRI showed a defect of the lateral femoral condyle while no cartilage loose body was clearly detected; (2) When the third MRI examination showed two loose bodies, which were difficult to distinguish from the infra-patellar fat pad and synovial wrinkle, additional sagittal PDWI sequence of 2mm slice thickness without fat suppression was performed. Nonetheless, the study had a few limitations: (1) thinner-layer MRI sequences available for Three-dimensional (3D) reconstruction were not performed because of equipment constraints [18, 19] and (2) due to patient variability in pain tolerance and cooperation during MRI imaging, the image qualities may have been affected, and using MRI to follow up on joint loose bodies is very expensive and not inefficient.

## CONCLUSION

MRI plays an important role in the grading of knee OCD. When cartilage fracture is identified while no cartilage loose body is clearly detected, short-term MRI follow-up is necessary, and a thin-layer PDWI sequence without fat suppression can be added to improve the detection of cartilage fragments.

## Figures and Tables

**Table 1:** MRI scan sequence and parameters of the 1^st^ knee examination.

	Sagittal T1WI	Sagittal PDWI-fs	Axial PDWI-fs	Coronal PDWI-fs
TR	320 ms	3190 ms	2860 ms	2700 ms
TE	11 ms	35 ms	43 ms	31 ms
Echo Spacing	10.9 ms	11.5 ms	7.16 ms	10.3 ms
FOV	160 ×160 ×72 mm	160 ×160 ×72 mm	160 ×160 ×120 mm	160 ×160 ×84 mm
Voxel	0.2 × 0.3 × 3.0 mm	0.5 × 0.5 × 3.0 mm	0.6 × 0.6 × 4.0 mm	0.6 × 0.6 × 3.5 mm
Slice thickness	3 mm	3 mm	4 mm	3.5 mm
Flip angle	150°	150°	150°	150°
NSA	1	1	1	1
Acquisition time	2min23s	2min30s	2min16s	2min23s

**Note:** T1WI = T1-weighted image. PDWI-fs = fat-suppressed proton density weighted image.TR = repetition time. TE = echo time. FOV = field of view. NSA = number of signal averaged.

## Data Availability

The data and supportive information are available within the article.

## LIST OF ABBREVIATIONS

OCD: Osteochondritis Dissecans
MRI: Magnetic Resonance Imaging
T1WI: T1-weighted Image
PDWI-fs: Fat-suppressed Proton Density-weighted Image
MFS: Microfractures

## STANDARDS OF REPORTING

CARE guidelines were followed.
